# DFT Exploration of a Pd-Doped InSe Monolayer as a Novel Gas Sensing Candidate upon SF_6_ Decomposition: SO_2_, SOF_2,_ and SO_2_F_2_

**DOI:** 10.3390/s25134156

**Published:** 2025-07-03

**Authors:** Xu Yang, Hao Cui, Zhongchao Liu, Yun Liu

**Affiliations:** 1 School of Intelligent Manufacturing, Nanyang Institute of Technology, Nanyang 473004, China; yangxuican@bjtu.edu.cn (X.Y.); fly200578@sina.com (Z.L.); 2 College of Artificial Intelligence, Southwest University, Chongqing 400715, China

**Keywords:** Pd-InSe monolayer, DFT, gas sensor, SF_6_ decomposition

## Abstract

Monitoring SF_6_ decomposition gases has emerged as a vital diagnostic technique for evaluating insulation conditions and identifying faults in SF_6_-based electrical equipment. This study comprehensively explores the adsorption properties and sensing capabilities of a Pd-doped InSe (Pd-InSe) monolayer for SF_6_ decomposition gases, including SO_2_, SOF_2_, and SO_2_F_2_, through density functional theory calculations. The Pd-InSe monolayer is constructed by substituting one Se atom with a Pd atom in the pristine InSe structure. Then, the Pd doping effect on the InSe monolayer and the adsorption behaviors of the Pd-InSe monolayer for three gases are thoroughly examined. The adsorption configurations, charge density differences, and electron localization functions are scrutinized to elucidate the gas adsorption mechanisms of the Pd-InSe monolayer; and the band structures, along with the density of states, are analyzed to gain insights into the resistive gas sensing mechanisms for detecting these three gases. Finally, the temperature-dependent recovery characteristics are evaluated to assess the reusability of the monolayer. These findings not only underscore the potential of the Pd-InSe monolayer for sensing SF_6_ decomposition gases but also open new avenues for the development of next-generation 2D materials in gas sensing applications within the field of electrical engineering.

## 1. Introduction

In high-voltage electrical systems, sulfur hexafluoride (SF_6_) serves as a critical insulating medium, particularly in gas-insulated switchgears (GISs) and gas-insulated transmission lines (GILs), owing to its exceptional arc-quenching capabilities and dielectric strength [1]. Consequently, the operational integrity and safety of these systems hinge on the stability and performance of SF_6_ gas. Despite its widespread use, prolonged exposure to electrical stresses in SF_6_-insulated equipment can lead to insulation degradation. Under conditions such as partial discharges, sparking, and thermal faults, SF_6_ undergoes dissociation, producing unstable low-fluorine sulfides (SF_x_, where x ranges from 1 to 5) and free fluorine atoms [2]. These decomposition processes are exacerbated further by trace contaminants like moisture and oxygen, initiating complex chemical pathways that generate highly corrosive and toxic byproducts—including SOF_2_, SO_2_F_2_, HF, H_2_S, and SO_2_ [3,4,5]. The gradual buildup of these compounds not only compromises the insulating properties of SF_6_ but also poses severe risks to equipment’s longevity and the grid’s reliability. Given these challenges, monitoring SF_6_ decomposition gases has emerged as a vital diagnostic tool for evaluating insulation conditions and preemptively identifying faults in electrical apparatus [6,7,8]. As the power industry advances, the development of precise detection methodologies and refinement of the existing analytical techniques remain imperative to ensuring compliance with stringent safety standards and sustaining the resilience of modern power infrastructure.

Recently, the III-IV chalcogenide family has emerged as a particularly promising class of materials for advanced gas sensing applications [9]. These semiconductors exhibit unique advantages including tunable band structures, exceptional charge carrier mobility, and pronounced surface reactivity [10]—properties that collectively guarantee highly sensitive and selective gas detection properties [11]. Particularly noteworthy is the two-dimensional InSe monolayer, whose exceptional surface-to-volume ratio and intrinsic electronic properties make it ideally suited to detecting trace-level SF_6_ decomposition byproducts with superior sensitivity. As reported, InSe exhibits outstanding electronic properties, including a high carrier mobility, a tunable bandgap, and strong surface activity [12]. These properties make it a compelling candidate for next-generation gas sensors, enabling sensitive detection at the molecular level. Theoretical density functional theory (DFT) simulations have demonstrated that a pristine InSe monolayer exhibits high selectivity and acceptable recovery properties for target gases [13]. Furthermore, to amplify its gas sensing response further, metal modification has been explored as an effective strategy. For instance, DFT studies reveal that Ru- or Rh-modified InSe monolayers exhibit a significantly enhanced gas adsorption energy and charge redistribution for NH_3_, NO_2_, and SO_2_ due to the catalytic and electronic effects of these two noble metal dopants [14]. Equally, Huihui Xiong et al. have reported applying a Ni-modified InSe monolayer to toxic gas species, revealing its potential as an outstanding gas sensor for detecting SO_2_ at room temperature [15]. These findings underscore the critical role and effectiveness of metal modification, especially using noble metals, in optimizing InSe’s surface reactivity and electronic coupling with gas molecules, thus boosting its adsorption and sensing capabilities.

Palladium (Pd), a noble metal renowned for its chemical stability and corrosion resistance, is widely employed as a surface dopant in nanomaterial systems. Studies demonstrate that Pd doping can significantly improve the gas sensing performance of TMDs such as WSe_2_ [16], MoS_2_ [17], and ZrS_2_ [18]. Similarly, Pd-decorated InSe monolayers have shown promise in detecting toxic gases (CO, NO, NH_3_, and H_2_S) through chemical adsorption [19]. However, surface-adsorbed Pd atoms may induce substantial structural deformation in the InSe monolayer, almost in the same plane as the atomic Se layer, creating stability concerns. To address this limitation, we propose substituting a Se atom with a Pd atom to create a Pd-doped InSe (Pd-InSe) monolayer. This doping strategy aims to preserve its structural integrity while enhancing its gas sensitivity. Using DFT calculations, in this work, we systematically evaluate the material’s potential to detect key SF_6_ decomposition products (SO_2_, SOF_2_, and SO_2_F_2_), in order to explore the potential of a Pd-InSe monolayer as an innovative gas sensing candidate [20]. These three gases exhibit distinct chemical behaviors and are widely adopted as key markers for SF_6_ insulation fault diagnosis in industry standards (e.g., IEC 60480) [21]. While other decomposition products (e.g., HF, H_2_S) are formed, their concentrations are typically orders of magnitude lower and their lifetimes shorter due to high reactivity. The selection of Pd is motivated by its proven catalytic properties and its ability to enhance the host material’s performance. Unlike surface decoration, lattice-incorporated Pd doping minimizes the structural disruption while potentially offering distinct gas–surface interaction mechanisms [22]. This unique doping strategy allows us to investigate the potential of the Pd-InSe monolayer as a highly sensitive and stable gas sensing material. The findings of this study can advance gas sensing technology by demonstrating the promising potential of InSe-based materials and elucidating the beneficial effects of doping monolayers with noble metals, paving the way for next-generation sensor development.

## 2. The Computational Details

Our computational methodology employed the DMol^3^ package [23], implementing the PBE-GGA (Perdew–Burke–Ernzerhof and generalized gradient approximation) functional to accurately describe the electron exchange–correlation effects [24]. To account for weak inter-molecular interactions, we integrated Tkatchenko and Scheffler (TS)’s dispersion correction scheme [25], which accounts for the crucial effects of anisotropic polarizability in layered materials with optimal accuracy. Equally, TS’s method outperforms D3 in describing the adsorption energetics of polar gas molecules (e.g., SOF_2_) due to its self-consistent treatment of charge density dependence while avoiding the heavy computational cost of MBD methods. Additionally, the calculations utilized a double numerical plus polarization (DNP) basis set for orbital expansion [26], with relativistic effects for metal atoms treated using the DFT semi-core pseudopotential (DSSP) approach [27]. Structural relaxation and electronic property computations were performed using a 10 × 10 × 1 Monkhorst–Pack *k*-point sampling [28]. Stringent convergence criteria were applied—energy (10^−5^ Ha), forces (2 × 10^−3^ Ha/Å), and displacement (5 × 10^−3^ Å) [29]—to ensure accuracy in this work. To analyze the electronic structure, we implemented additional precision parameters including a self-consistent field tolerance of 10^−6^ Ha, a 5.0 Å orbital cutoff radius, and a 0.005 Ha smearing width [30,31].

Our computational framework adopted electron volts (eV) as the standard energy unit, with corresponding conversion factors (1 eV = 23.06 kcal/mol = 96.48 kJ/mol) included for comparative analysis [32]. The simulation system comprised an expanded 4 × 4 × 1 InSe monolayer supercell (32 In and 32 Se atoms), incorporating a 20 Å vacuum to eliminate periodic boundary effects [33]. For electronic characterization, we implemented the Hirshfeld population analysis, a methodology particularly valuable for its parameter-independent nature, to quantify both the atomic charge of the Pd dopant (*Q*_Pd_) and the interfacial charge transfer during gas adsorption (*Q*_T_) [34]. Spin-polarized calculations were systematically performed throughout all simulation stages to account for electron spin effects [35].

## 3. Results and Discussion

### 3.1. Pd Doping Properties Within the InSe Monolayer

**Figure 1:**
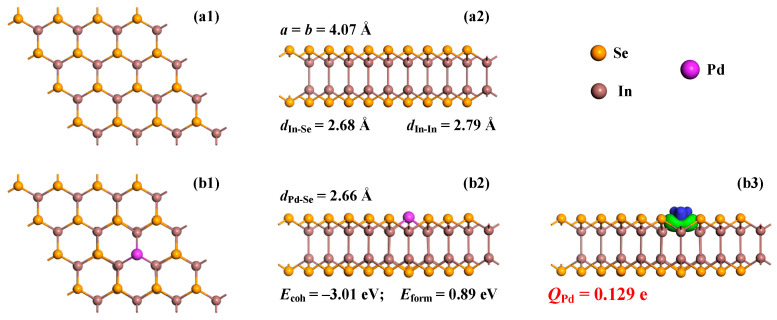
Configurations and CDDs of (**a1**,**a2**) pristine and (**b1**–**b3**) Pd-doped InSe monolayers. In CDD, the green areas are electron accumulations, and the blue areas are electron depletions with an isosurface of 0.02 e/Å^3^.

Firstly, geometric optimizations are performed to obtain the optimized structures of the pristine and Pd-doped InSe monolayers. Then, a comparative analysis of the geometric and electronic properties between the two monolayers is deemed significant, as it highlights the role of Pd doping in modifying the InSe monolayer’s intrinsic properties, which is advantageous for potential gas sensing applications. Since the Se atoms occupy the outermost layer in the InSe lattice, one can assume that substituting Se atoms, rather than In atoms, is expected to be more advantageous for surface modification to enhance the interaction between the doped atoms and the gas species due to greater accessibility and electronic coupling at the surface. Therefore, the Pd-InSe monolayer in this theoretical study is modeled by replacing a Se atom within the InSe monolayer with a Pd atom, and the cohesive energy (*E*_coh_) and the formation energy (*E*_form_) are defined to evaluate the geometric stability and the energy required to establish such a configuration. *E*_coh_ is calculated by [36]

(1) $$E_{\mathrm{coh}}=\frac{E_{\mathrm{Pd}-\mathrm{InSe}}-E_{\mathrm{Pd}}-32\times E_{\mathrm{In}}-31\times E_{\mathrm{Se}}}{64}$$

in which $E_{\mathrm{Pd}-\mathrm{InSe}}$ is the total energy of the Pd-InSe monolayer, and *E*_Pd_, *E*_In_, and *E*_Se_ are the energies of the isolated Pd, In, and Se atoms, respectively. *E*_form_ is expressed as [37]

(2) $$E_{\mathrm{form}}=E_{\mathrm{Pd}-\mathrm{InSe}}-E_{\mathrm{InSe}}-\mu_{\mathrm{Pd}}+\mu_{\mathrm{Se}}$$

wherein $E_{\mathrm{Pd}-\mathrm{InSe}}$ and $E_{\mathrm{InSe}}$, respectively, indicate the energy of the Pd-doped and pristine InSe monolayers, while *μ*_Pd_ and *μ*_Se_, respectively, indicate the chemical potential of single Pd and Se atoms in their bulk forms. With all of these values and configurations, the configurations of the pristine and Pd-doped InSe monolayers, as well as the charge density difference (CDD) of the Pd-InSe monolayer, are plotted in Figure 1.

The optimized geometries of the pristine InSe monolayer, depicted in Figure 1a1,a2, exhibit a lattice constant of *a* = *b* = 4.07 Å, as well as an In-Se bond length of 2.68 Å and an In-In bond length of 2.79 Å. These parameters are very close to those in the previous reports [38,39], in which the lattice constant of the pristine InSe monolayer was calculated to be 4.05 and 4.06 Å, respectively. Such minor differences in this parameter can be attributed to the use of different basic sets in various theoretical calculations [40], underscoring the high accuracy of the current computational approach. The optimized configurations of the Pd-InSe monolayer are displayed in Figure 1b1,b2. Notably, the incorporation of the Pd dopant induces minimal lattice distortion, with the three equivalent Pd-Se bonds measuring 2.66 Å, only 0.75% shorter than the In-Se bonds (2.68 Å) in the pristine monolayer. This remarkable structural preservation stems from the comparable ionic radii of Pd^2+^ (0.86 Å) and In^3+^ (0.80 Å), which enable nearly isostructural doping without compromising the host framework [41]. This structural compatibility is crucial to achieving efficient doping and maintaining the overall structural integrity of the material, as indicated by the stable integration of the dopant atom into the lattice structure [42]. In the meanwhile, a vibrational analysis of the Pd-InSe monolayer is calculated using the finite displacement method within the DMol^3^ module, where the Hessian matrix is constructed via numerical differentiation of the atomic forces (±0.015 Å displacements) in Cartesian directions. Accordingly, the system’s exceptional stability is quantitatively demonstrated through multiple thermodynamic metrics: (i) *E*_coh_ of −3.01 eV/atom, reflecting strong inter-atomic bonding within the Pd-InSe lattice [43]; (ii) a positive *E*_form_ value of 0.89 eV, indicating the requirement for an external energy input in the doping process, a property that actually enhances the operational stability under ambient conditions [44]; and (iii) the absence of imaginary frequencies in phonon spectra (22.57~630.19 cm^−1^), confirming dynamic stability and ruling out structural instabilities [45]. These comprehensive stability metrics collectively verify the structural integrity of the Pd-InSe monolayer. The slight bond contraction actually can benefit the gas sensing performance by modulating the electronic structure without causing lattice defects [46], a critical advantage for reliable sensor operation. The positive *E*_form_ value further suggests that the doped configuration will remain stable during sensing applications, as the energy barrier prevents spontaneous dopant migration or desorption.

The analysis of the charge density difference (CDD) of the Pd-InSe monolayer reveals pronounced electron accumulation localized at the Pd-Se interfacial regions, as revealed in Figure 1b3. This indicates strong orbital hybridization and covalent interaction between the Pd and Se atoms, which enhances the bonding strength and structural stability of the doped system [47]. Furthermore, the Hirshfeld analysis demonstrates that the Pd dopant exhibits an effective positive charge of +0.129 e, confirming its electron-donating behavior within the InSe lattice. Correspondingly, the CDD map shows significant electron depletion around the Pd site, consistent with its role as an electron donor. Notably, this charge redistribution differs markedly from that in the pristine InSe monolayer, where the corresponding Se atom carries a negative charge of −0.162 e. The transition from an electron-rich Se site to an electron-deficient Pd center induces substantial modifications in the local electronic environment. Thus, the substitutional incorporation of a Pd atom can induce significant modifications to the intrinsic electronic structure of the InSe monolayer.

**Figure 2:**
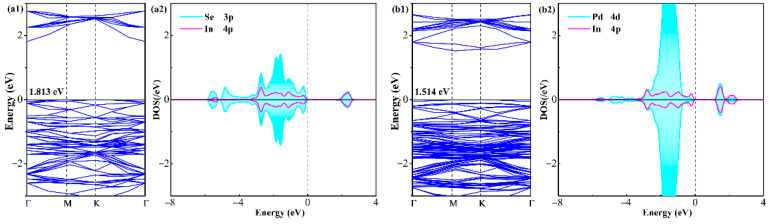
BSs and atomic DOS of (**a1**,**a2**) pristine and (**b1**,**b2**) Pd-doped InSe monolayers.

To elucidate the profound influence of Pd doping on the electronic properties of the InSe monolayer, we present a comprehensive analysis of the band structures (BSs) and density of states (DOS) for both pristine and Pd-doped systems in Figure 2. The pristine InSe monolayer exhibits an indirect bandgap of 1.813 eV in our calculations, with the valence band maximum (VBM) located at the K point and the conduction band minimum (CBM) at the Γ point. This result shows excellent agreement with previous theoretical reports of 1.968 eV [19], validating the reliability of our computational methodology based on the PBE functional. Therefore, in the following sections, we entirely adopt the PBE functional to calculate the electronic properties of the analyzed systems.

In contrast, the bandgap of the Pd-InSe monolayer narrows to 1.514 eV, representing a 16.5% reduction that enhances the conductivity by facilitating electron excitation across the energy barrier [48]. Equally, considering the strong relativistic effects of heavy elements like Pd, the spin–orbit coupling (SOC) effect becomes significant to the analysis in terms of bandgap modulation. For the Pd-InSe monolayer, our DFT calculations indeed show modest SOC-induced band splitting (~50 meV) near the valence band maximum, which is much smaller than the Pd-induced bandgap-narrowing mechanism (about a 0.3 eV reduction). Therefore, the effect of SOC in this system exerts little impact on the bandgap modulation. In the meanwhile, the valence and conduction band extrema of the Pd-InSe monolayer remain at distinct *k*-points, revealing that although the substituted Pd dopant impacts the bandgap, it does not deform the bandgap character of the pristine InSe system. Crucially, the doped system shows a substantial increase in the DOS near the Fermi level and additional electronic transition pathways, which collectively enhance the surface chemical reactivity and charge transfer kinetics in the gas interactions [49]. These properties are favorable for obtaining faster response times and higher sensitivity in a gas sensor. These results demonstrate that strategic Pd doping serves as an effective approach to engineering the electronic properties of InSe monolayers while maintaining their structural integrity, offering new opportunities to develop advanced sensing devices with tunable performance characteristics.

A detailed examination of the atomic DOS for both the pristine and Pd-doped InSe systems reveals critical insights into their bonding characteristics and electronic interactions. In the pristine monolayer, symmetric spin-up and spin-down configurations confirm the non-magnetic nature of the material, a property preserved upon Pd doping. The atomic DOS analysis demonstrates strong orbital hybridization between the Se 4p and In 4p orbitals at distinct energy levels (−5.4 eV, −3.2 to −0.2 eV, and 2.3 eV), highlighting the covalent character of the In-Se bonds in the pristine system [50]. Remarkably, the incorporation of Pd introduces new hybridization features between the Pd 4d and In 4p orbitals near the Fermi level (−5.4 eV, −2.8 to −0.2 eV, 1.5 eV, and 2.2 eV), indicating significant electronic restructuring while maintaining robust bonding interactions. These orbital mixing patterns not only confirm the formation of stable Pd-Se bonds but also reveal how the dopant modifies the local electronic environment without disrupting the overall structural integrity. The enhanced orbital interactions near the Fermi level in the doped system are particularly noteworthy, as they suggest improved charge delocalization and chemical reactivity, key factors for potential sensing applications. This comprehensive DOS analysis provides valuable guidance for designing advanced functional materials with tailored properties, highlighting the impact of Pd doping on the electronic properties of InSe material.

### 3.2. Gas Adsorption in the Pd-InSe Monolayer

The investigations now focus on the SF_6_ decomposition product (SO_2_, SOF_2_, and SO_2_F_2_) adsorption behavior of the Pd-InSe monolayer. Adsorption studies are conducted by strategically positioning each target gas molecule at multiple orientations within the Pd-InSe lattice, keeping an initial adsorption distance of 2.5 Å from the active Pd site. It is worth mentioning that such a distance is carefully selected to correspond to the typical range of van der Waals interactions (2–3 Å) between gas molecules and solid surfaces [51]. This distance reflects a balance between covalent coordination (short-range) and vdW interactions (long-range), as validated using our TS-corrected DFT calculations. To quantitatively assess the adsorption strength and stability, we employ calculations of the adsorption energy (*E*_ad_) using the established formula [52]:

(3) $$E_{\mathrm{ad}}=E_{\mathrm{Pd}-\mathrm{InSe}/\mathrm{gas}}-E_{\mathrm{Pd}-\mathrm{InSe}}-E_{\mathrm{gas}}$$

in which $E_{\mathrm{Pd}-\mathrm{InSe}/\mathrm{gas}}$ and $E_{\mathrm{gas}}$ represent the total energy of the adsorption complex and the isolated gas molecule, respectively. Through screening various adsorption configurations, we identify the most stable configuration (MSC) for each gas–surface system based on the most negative *E*_ad_ value, which reveals the preferred molecular orientation and binding mechanism. This systematic approach not only elucidates the fundamental adsorption characteristics but also provides crucial insights into the charge transfer dynamics induced by gas adsorption, enabling a thorough understanding of the gas–surface interactions at the atomic level.

**Figure 3:**
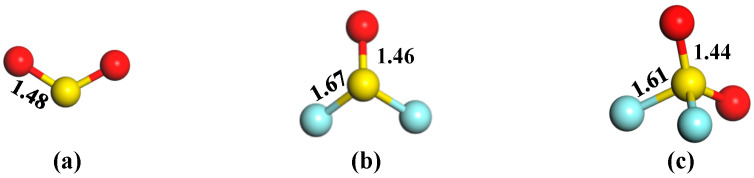
Morphologies of three SF_6_ decomposition gases. (**a**) SO_2_, (**b**) SOF_2_, and (**c**) SO_2_F_2_.

Figure 3 illustrates the optimized morphologies of three SF_6_ decomposition gases, namely SO_2_, SOF_2_, and SO_2_F_2_, exhibiting distinct structural characteristics. The SO_2_ molecule adopts a bent V-shaped geometry with O-S-O bond angles of 119.9° and two equivalent S=O double bonds measuring 1.48 Å. SOF_2_ displays a trigonal pyramidal structure where the S=O double bond (1.46 Å) is shorter than the two S-F single bonds (1.67 Å), with F-S-F and O-S-F angles of 93.2° and 107.2°, respectively. In contrast, SO_2_F_2_ features a tetrahedral arrangement with two S=O bonds (1.44 Å) and two S-F bonds (1.61 Å), with the following angles: O-S-O: 126.7°; F-S-F: 94.4°; and O-S-F: 107.8°. These molecular parameters are in good agreement with the previous report [23], indicating the good accuracy of this work.

**Figure 4:**
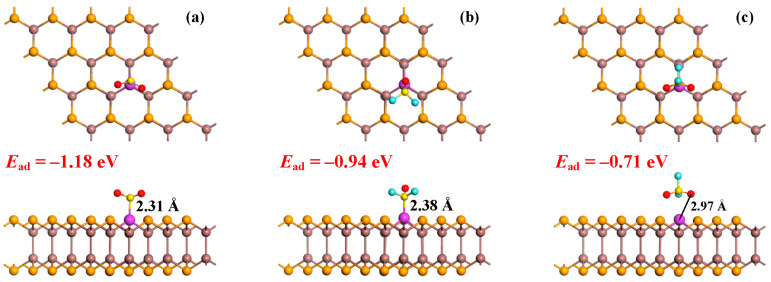
MSC of top and side views for adsorption of three SF_6_ decomposition gases onto Pd-InSe monolayer. (**a**) SO_2_ system, (**b**) SOF_2_ system and (**c**) SO_2_F_2_ system.

Figure 4 presents the MSC for the three SF_6_ decomposition products (SO_2_, SOF_2_, and SO_2_F_2_) on the Pd-InSe monolayer. They are not only identified according to the configuration with the lowest *E*_ad_ value but also confirmed through a vibrational analysis without the imaginary frequency. For SO_2_ adsorption, our investigations consider three distinct orientations: a molecule-parallel configuration, a S-atom-oriented configuration, and an O-atom-oriented configuration. After thorough geometric optimization, the system preferentially adopts the S-atom-oriented position wherein the SO_2_ molecule is vertically aligned and the S atom forms a strong chemical bond with the Pd dopant. The measured Pd-S bond length of 2.31 Å, combined with the significant adsorption energy of −1.18 eV, clearly demonstrates chemisorption behavior [53]. In the case of SOF_2_ adsorption, we examined two primary configurations: the S-atom orientation and the F-O-F plane orientation. The optimized structure reveals that the molecule preferentially binds through the S-atom oriented configuration, forming a Pd-S bond of 2.38 Å with an *E*_ad_ value of −0.94 eV. While still indicating chemisorption and confirming the formation of a stable chemical bond, the slightly longer bond length and reduced *E*_ad_ value (compared to these values for SO_2_) suggest a moderately weaker interaction. The adsorption behavior of SO_2_F_2_ presents a markedly different scenario. Despite evaluating multiple initial configurations (O-F-O and F-O-F orientations), the optimized structure shows no evidence of chemical bond formation. The shortest observed Pd-O distance of 2.97 Å significantly exceeds the sum of the covalent radii (1.85 Å) [54], while the calculated *E*_ad_ value of −0.71 eV falls within the physisorption range [55].

These results demonstrate a clear hierarchy in the adsorption strength: SO_2_ (−1.18 eV) > SOF_2_ (−0.94 eV) > SO_2_F_2_ (−0.71 eV). The progressive weakening of the interactions correlates with increasing substitution of fluorine into the molecule series, which affects both molecular polarity and orbital accessibility. From an application perspective, the system’s ability to strongly chemisorb SO_2_ while only weakly physisorbing SO_2_F_2_, with SOF_2_ showing intermediate behavior, provides an excellent physical basis for developing sensitive and selective gas detection platforms. Furthermore, the significant differences in *E*_ad_ (ΔE = 0.47 eV between extreme cases) suggest that temperature-modulated desorption could potentially be employed to discriminate between these decomposition products in practical sensing applications.

**Figure 5:**
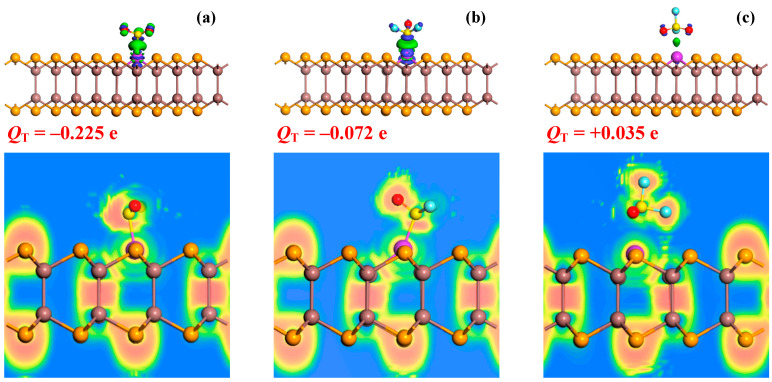
CDDs and ELFs of (**a**) the SO_2_ system, (**b**) the SOF_2_ system, and (**c**) the SO_2_F_2_ system. For the CDD, the isosurface is set as 0.02 e/Å^3^, and for the ELF, the value ranges from 0 to 1.

Figure 5 presents the CDD and electron localization function (ELF) analyses for the three gas-adsorbed systems, providing fundamental insights into the charge transfer mechanisms and bonding nature between the SF_6_ decomposition gases and the Pd-InSe monolayer. The CDD distributions reveal pronounced electron accumulation regions at the Pd-S interfaces in both the SO_2_ and SOF_2_ systems, demonstrating significant electron cloud overlap and a strong covalent character for these bonds. This substantial charge redistribution confirms the formation of stable chemical bonds, consistent with their large negative *E*_ad_. In striking contrast, the SO_2_F_2_ system shows minimal electron accumulation between Pd and O/S atoms, with only diffuse charge redistribution observed, clearly indicating the absence of strong chemical bonding and supporting its physisorption behavior. Moreover, a detailed examination of the CDD patterns reveals distinct behaviors among the three gas molecules. Both SO_2_ and SOF_2_ demonstrate pronounced electron accumulation regions surrounding their molecular structures, indicating strong electron-accepting characteristics. This electron-rich environment facilitates effective charge transfer from the Pd-InSe monolayer to the adsorbed molecules. In striking contrast, the SO_2_F_2_ molecule is surrounded by obvious electron depletion, suggesting an electron-donating propensity. The quantitative Hirshfeld charge analysis substantiates these observations with precise *Q*_T_ values of −0.225 e (SO_2_), −0.072 e (SOF_2_), and +0.035 e (SO_2_F_2_). The negative *Q*_T_ values for SO_2_ and SOF_2_ confirm their electron-accepting nature, with SO_2_ showing an approximately three times greater charge acceptance than that of SOF_2_. The positive *Q*_T_ value for SO_2_F_2_ uniquely identifies its electron-donating behavior. This systematic variation in the charge transfer characteristics establishes a clear and physically meaningful hierarchy: SO_2_ > SOF_2_ > SO_2_F_2_. Remarkably, the *Q*_T_ hierarchy exhibits a perfect correlation with the determined adsorption strength trends, providing a unified understanding of the adsorption phenomena.

Additionally, the ELF analysis provides additional compelling evidence for the bonding nature in these systems. For SO_2_ and SOF_2_ adsorption, the ELF values near the Pd-S bonds exceed 0.5, with localized electron pairs clearly visible between the atoms, a definitive signature of covalent bond formation [56]. The SO_2_ system shows particularly high ELF values (~0.6), indicating its stronger covalent character compared to that for SOF_2_ (~0.5), consistent with their respective *E*_ad_ values. In stark contrast, the SO_2_F_2_ system displays uniformly low ELF values (~0.2) across the Pd-O/S interface, confirming the absence of covalent bonding and supporting the physisorption mechanism [57]. The distinct electronic signatures revealed by the CDD and ELF analyses support the notably strong binding affinity of the Pd-InSe monolayer towards the SO_2_ and SOF_2_ molecules, indicative of a chemisorptive interaction, while relatively weaker physisorption occurs for the SO_2_F_2_ molecule.

### 3.3. Modulated Electronic Properties in Gas Adsorption

**Figure 6:**
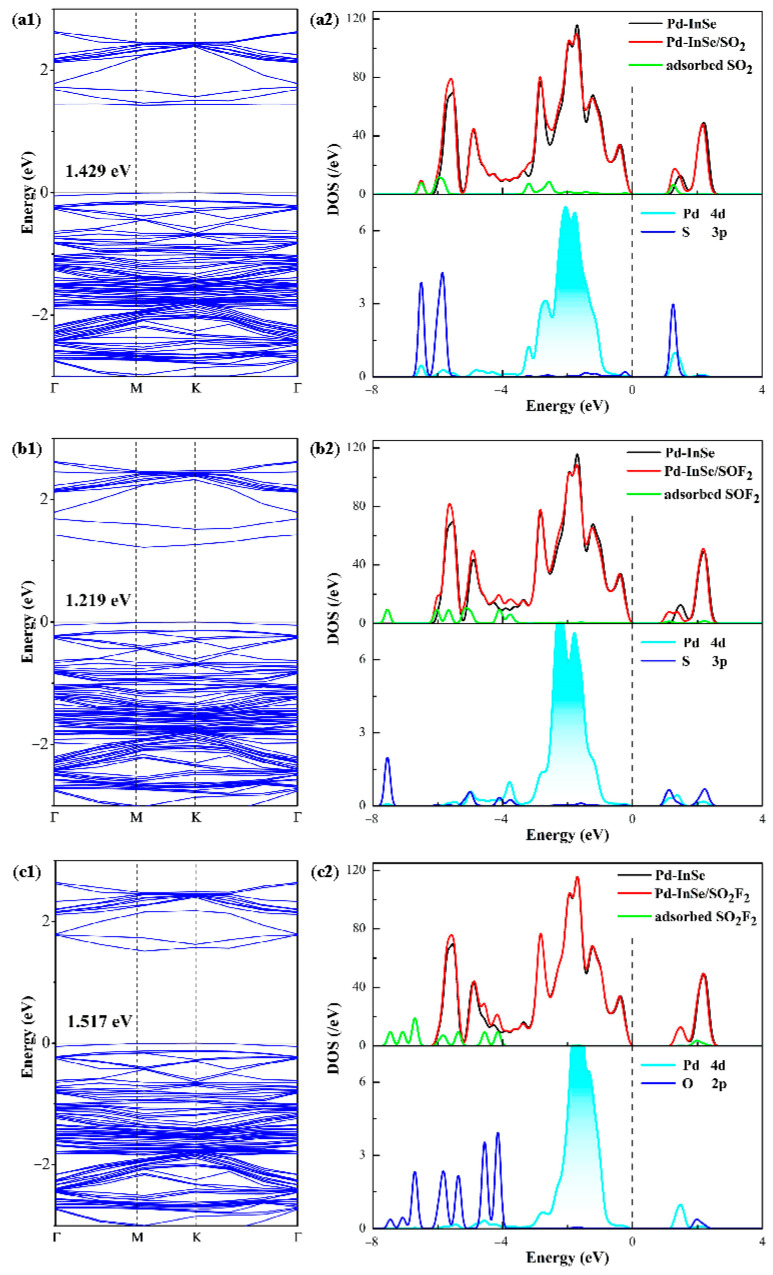
BSs and DOS of (**a1**,**a2**) SO_2_ system, (**b1**,**b2**) SOF_2_ system, and (**c1**,**c2**) SO_2_F_2_ system.

A comprehensive analysis of the BSs and DOS for the Pd-InSe monolayer upon interaction with the SF_6_ decomposition products is presented in Figure 6, revealing significant insights into the electronic modulation induced by gas adsorption. Exhibiting an indirect bandgap of 1.514 eV, the Pd-InSe monolayer undergoes distinct modifications depending on the adsorbed gas species. Most notably, SO_2_ adsorption reduces the bandgap to 1.429 eV, while SOF_2_ causes an even more substantial reduction to 1.219 eV, representing decreases of 5.6% and 19.5%, respectively, in comparison to the isolated system. These bandgap-narrowing effects are accompanied by the emergence of well-defined impurity states within the original bandgap region, originating from the adsorbed SO_2_ molecule at 1.2 eV and the adsorbed SOF_2_ molecule at 1.1 eV. The stronger bandgap reduction and more prominent impurity states in the SOF_2_ system suggest its potential to induce a more significant sensing response compared to SO_2_ [58]. In contrast, SO_2_F_2_ adsorption results in a fundamentally different electronic response, with the bandgap slightly increasing to 1.517 eV (a 0.2% change) and showing minimal impurity state formation near the Fermi level. This electronic behavior correlates precisely with the weak physisorption characteristics observed in the previous analyses, where the lack of strong chemical bonding prevents significant electronic coupling between the molecule and the monolayer. The marginal bandgap variation and the absence of notable in-gap states explain the poor sensing response predicted for SO_2_F_2_.

A thorough examination of the total DOS before and after the adsorption of gas by the Pd-InSe monolayer reveals significant alterations in the electronic structure, particularly in the vicinity of the Fermi level. In the SO_2_-adsorbed system, we observe the appearance of distinct new states at −2.7, −1.4, −1.1, −0.2, and 1.2 eV. Similarly, the SOF_2_-adsorbed system exhibits characteristic new states at −3.8, −1.5, −0.3, 1.1, and 2.2 eV. These newly formed states near the Fermi level, from the state contributions of the adsorbed SO_2_ and SOF_2_ molecules, play a crucial role in modifying the monolayer’s electronic properties, with certain states effectively filling the original bandgap region and consequently reducing the system’s bandgap [51]. A detailed orbital DOS analysis provides deeper insights into the interactions’ nature. The S 3p orbitals from both the SO_2_ and SOF_2_ molecules show substantial overlap with the Pd 4d orbitals, creating strong hybridization peaks in the energy range between −2.5 eV and 1.5 eV. This orbital mixing not only facilitates the formation of stable Pd-S chemical bonds but also serves as direct evidence of the strong electronic coupling at the adsorption interface. These findings offer compelling evidence for the chemisorption mechanism, as the extensive orbital hybridization and new state formation clearly demonstrate covalent bond characteristics rather than weak physical interactions [59].

On the other hand, the total DOS analysis reveals only minimal state contributions from the adsorbed SO_2_F_2_ molecule, appearing weakly at −1.6 eV and 2.0 eV in the energy spectrum. Crucially, the electronic states near the Fermi level remain virtually unchanged compared to those in the isolated Pd-InSe monolayer, without in-gap states or band edge modifications, reflecting the weak physical interaction between SO_2_F_2_ and the monolayer. The orbital DOS analysis shows only modest hybridization between the Pd 4d and O 2p orbitals. The limited orbital overlap and the absence of strong bonding states suggest that any interaction occurs primarily through weak van der Waals forces rather than chemical bonding [60]. All of these observed electronic behaviors correlate well with the previously adsorption characteristic analyses, providing a consistent picture of the adsorption process across multiple characterization methods.

The practical implications of these electronic structure characteristics are significant for gas sensing applications. The negligible bandgap modification and the lack of substantial new states near the Fermi level suggest that the Pd-InSe monolayer exhibits a minimal electrical response to SO_2_F_2_ exposure. This stands in direct contrast to the pronounced responses predicted for SO_2_ and SOF_2_, highlighting the monolayer’s potential for selective gas detection. These insights provide valuable information for the potential application of Pd-InSe monolayers in gas sensing and other related fields.

### 3.4. Gas Sensing Exploration

**Figure 7:**
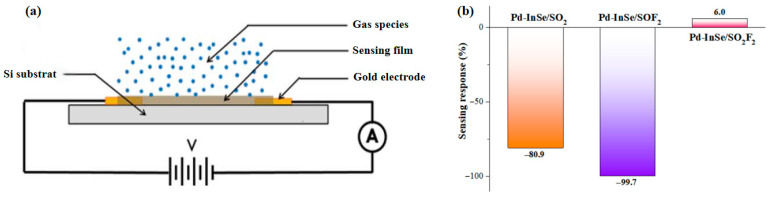
(**a**) Sensor architecture and (**b**) sensing performances of Pd-InSe monolayer.

Based on the comprehensive analysis of its adsorption characteristics and electronic properties, the Pd-InSe monolayer demonstrates exceptional potential as a resistive-type gas sensor for detecting these three SF_6_ decomposition gases, particularly showing superior sensitivity toward SO_2_ and SOF_2_. This section systematically investigates the fundamental sensing mechanism and quantitatively evaluates the detection capability, which is paramount for real-time monitoring of SF6-insulated electrical equipment. The working principle of this resistive gas sensor relies on measurable changes in the electrical resistance when the sensing material interacts with the target gas molecules. This phenomenon originates from the direct correlation between a material’s band structure and its conductivity properties. Specifically, the adsorption-induced bandgap modification (Δ*B*_g_) alters the charge carrier concentration and mobility significantly, thereby affecting the overall conductivity. Our theoretical framework establishes that the electrical conductivity (*σ*) follows an exponential relationship with the bandgap [61]:

(4) $$\sigma =A\cdot e^{\left(-B_{g}/2kT\right)}$$

wherein A represents a material-specific proportionality constant, *B_g_* denotes the bandgap of the material, *k* is the Boltzmann constant (8.318 × 10^−3^ kJ/(mol·K)), and *T* is the operating temperature. This relationship highlights that even a modest bandgap reduction can substantially enhance the electrical conductivity due to the exponential dependence. Furthermore, the sensing response (*S*) can be quantitatively expressed as

(5) $$S=\frac{\sigma_{\mathrm{gas}}{-}^{1}-\sigma_{\mathrm{pure}}{-}^{1}}{\sigma_{\mathrm{pure}}{-}^{1}}{=e}^{-\Delta B_{g}/2kT}-1$$

wherein Δ*B*_g_ represents the change in bandgap between the gas-adsorbed and pristine systems. The developed theoretical framework enables quantitative predictions of the Pd-InSe monolayer’s sensing response through Equation (5), with Figure 7 illustrating both the sensor architecture and the sensing performances. The standard device configuration, shown in Figure 7a, employs interdigitated gold electrodes on a silicon/silicon oxide substrate, onto which the Pd-InSe thin film is deposited using chemical vapor deposition techniques [62,63]. The sensing mechanism relies on resistance measurements comparing the baseline state (in an inert atmosphere) with the gas-exposed conditions, where adsorption-induced charge transfer modulates the film’s conductivity. Our calculations reveal favorable response characteristics, as illustrated in Figure 7b: the monolayer demonstrates a −80.9% response to SO_2_ and remarkably a −99.7% response to SOF_2_. These substantial negative responses correspond to conductivity increases of approximately 5× and 100, respectively, which can enable lower detection limits, faster response times and better signal-to-noise ratios for gas detection [64]. In contrast, the minimal 6.0% response to SO_2_F_2_ confirms effective discrimination of this interference species, consistent with its physisorption behavior and negligible electronic perturbation. These findings validate the Pd-InSe monolayer as a highly promising material for advanced gas sensors in real-world SF_6_ monitoring applications, where the key requirement is accurate identification of SOF_2_ as the primary decomposition marker.

**Figure 8:**
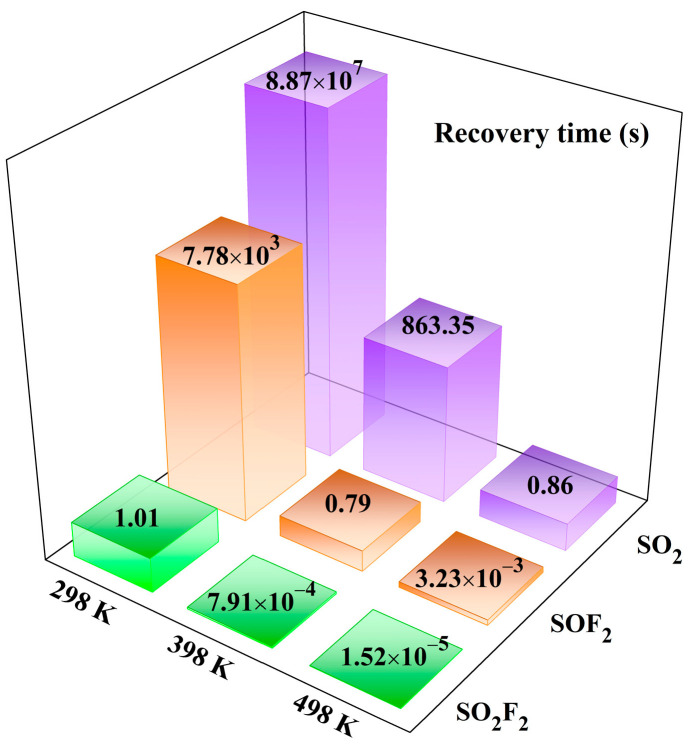
The recovery times of the Pd-InSe monolayer for the three SF_6_ decomposed species.

Additionally, the recovery time (τ), a critical parameter for evaluating the reusability of the sensing material in subsequent gas detection cycles, is defined as the time required for gas desorption from the nano-surfaces and is formulated as [65]

(6) $$\tau =A^{-1}e^{\left(-E_{\mathrm{ad}}/kT\right)}$$

where *A* is the attempt frequency of 10^12^ s^−1^ [66], *E*_ad_ is the adsorption energy, *k* is the Boltzmann constant, and *T* is the working temperature. This equation offers a method for calculating the recovery time of the gas sensing material. Based on Equation (6), the recovery times for SO_2_, SOF_2_, and SO_2_F_2_ of the Pd-InSe monolayer are depicted in Figure 8.

**Figure 9:**
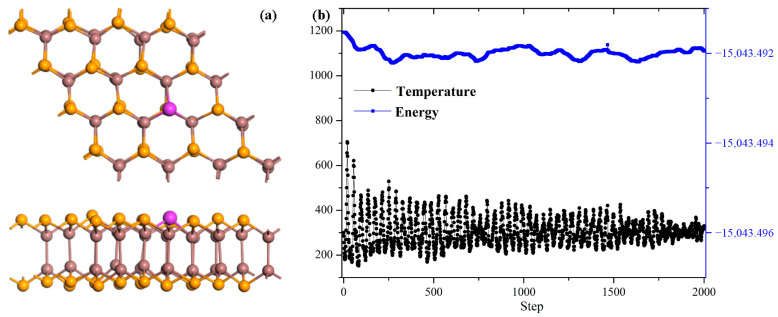
Molecular simulation results. (**a**) The structure of the Pd-InSe monolayer after simulation, and (**b**) energy and temperature fluctuations in the 2000 steps.

The desorption analysis exhibited in Figure 8 provides the operational characteristics of the Pd-InSe monolayer as a gas sensor, revealing its distinct temperature-dependent behaviors for the different SF_6_ decomposition gases. At ambient temperature (298 K), the system demonstrates vastly different recovery dynamics: SO_2_ exhibits an impractically long recovery time of 8.87 × 10^7^ s due to the strong chemisorption, while SOF_2_ shows a more moderate but still considerable recovery period of 7.78 × 10^3^ s. In contrast, SO_2_F_2_ displays instantaneous recovery (1.01 s), consistent with its weak physisorption behavior and the negligible sensing response. Equally, temperature modulation proves particularly effective for optimizing the SO_2_ and SOF_2_ desorption performance, where the recovery time becomes 0.79 s at 398 K for SOF_2_ and 0.86 s at 498 K for SO_2_. These findings enable near-real-time monitoring of these two gas species, suitable for reversible detection under practical conditions. In addition, effective temperature modulation requires excellent thermostability of the sensing material. To evaluate this, we perform molecular dynamics simulations of the Pd-InSe monolayer across a temperature range of 200–500 K for 2 ps with a time step of 1 fs, with the results obtained shown in Figure 9. From Figure 9a, while minor atomic displacements are observed within the Pd-InSe monolayer, these displacements are insufficient to cause lattice distortion. Furthermore, despite the temperature fluctuations in Figure 9b, the total energy of the supercell remains stable throughout the 2000 simulation steps. These results demonstrate that the Pd-InSe monolayer maintains excellent structural integrity under thermal stress, confirming its robust thermostability for gas sensor applications.

To sum up, the Pd-InSe monolayer emerges as a compelling candidate for a resistive gas sensor, demonstrating the high sensitivity and favorable reusability upon SO_2_ and SOF_2_ detection at 398 K, making it ideally suited to SF_6_ insulation monitoring. Specifically, the monolayer’s selectivity for SOF_2_ is particularly valuable for electrical equipment diagnostics, as SOF_2_ serves as a key indicator of the partial discharge while avoiding cross-sensitivity with other decomposition products. Importantly, heating should be applied to accelerate the recovery process, which can easily be achieved through direct Joule heating via the interdigital electrodes, which provides highly localized thermal activation while maintaining near-ambient conditions in the surrounding GIS environment. This electrode-based heating approach requires minimal power consumption (typically < 1 W) due to the nanoscale thickness of the sensing material, and the brief heating duration (<30 s per cycle) ensures a negligible impact on the overall GIS’s internal temperature. This approach is consistent with the existing heated gas sensor technologies used in industrial applications. The sensor’s compact design and pulsed operation mode minimize energy requirements further while maintaining its detection performance.

## 4. Conclusions

Through DFT calculations, this study demonstrates the exceptional potential of Pd-InSe monolayers as a promising sensing material for SF_6_ decomposition gases. The structurally stable Pd-InSe configuration, characterized by an *E*_coh_ value of −3.01 eV and an *E*_form_ value of 0.89 eV, exhibits distinct interaction mechanisms with different gas species: strong chemisorption for SO_2_ and SOF_2_ versus weak physisorption for SO_2_F_2_. These interactions induce significant modifications in the electronic structure, yielding bandgap reductions in the SOF_2_ and SO_2_ systems that translate into remarkable sensing responses of −99.7% and −80.9%, respectively, while SO_2_F_2_ shows a negligible response (6.0%). The monolayer’s operational versatility is evidenced by its temperature-dependent recovery characteristics: SOF_2_ detection achieves sub-second recovery (0.79 s) at moderately elevated temperatures (398 K), while SO_2_ monitoring requires higher temperatures (498 K) for a comparable performance. These findings not only advance our fundamental understanding of Pd-InSe monolayers as ideal candidates for practical SF_6_ insulation monitoring applications but also pave the way for designing next-generation systems for responding to specific gas molecules and monitoring electrical infrastructure.

## Data Availability

The data presented in this study are available on request from the corresponding author.
